# *In vivo* colonization profile study of *Bordetella bronchiseptica* in the nasal cavity

**DOI:** 10.1111/j.1574-6968.2007.00852.x

**Published:** 2007-08-22

**Authors:** Yasuhiko Irie, Ming H. Yuk

**Affiliations:** Department of Microbiology, University of Pennsylvania School of Medicine Philadelphia, PA, USA

**Keywords:** *Bordetella bronchiseptica*, nasal ciliated epithelia, bacterial colonization, filamentous hemagglutinin (FHA), Bvg^i^ phase

## Abstract

*Bordetella bronchiseptica* chronically infects a wide range of mammals, and resides primarily in the nasal cavity of the infected host. Multiple virulence factors of *Bordetella* species have been studied in the context of lower respiratory tract infections, but relatively less is known about the bacterial life cycle in the nasal cavity. Evidences were discovered for Bvg intermediate (Bvg^i^) phase expression *in vivo* and that the major adhesin filamentous hemagglutinin plays a major role in the colonization of *B. bronchiseptica* in the unciliated olfactory epithelia of the nasal cavity.

## Introduction

*Bordetella pertussis*, *Bordetella parapertussis* and *Bordetella bronchiseptica* are closely related Gram-negative species that colonize the upper respiratory tract of mammals and mainly cause pathogenic symptoms in the lower respiratory tract. *Bordetella pertussis* and most *B. parapertussis* strains only infect humans and generally cause acute respiratory diseases (Bjørnstad & Harvill, 2005). *Bordetella bronchiseptica* often causes persistent chronic infections in a wide range of mammalian host and colonizes the hosts for their lifetimes (Mattoo & Cherry, 2005). Despite the differences in host niches and symptoms due to infections by the *Bordetella* species, most virulence factors are highly conserved.

A majority of *Bordetella* virulence factors are transcriptionally controlled by a two-component system BvgAS (*Bordetella* virulence genes). The response regulator BvgA has different binding specificities to certain promoters of virulence factors depending on its phosphorylation status as modulated by the sensor histidine kinase BvgS (Cotter & Jones, 2003). In the virulent Bvg^+^ phase, toxins/toxin delivery systems such as pertussis toxin, adenylate cyclase/hemolysin bifunctional toxin (CyaA), dermonecrotic toxin, and type III secretion system are being expressed. Adhesins such as filamentous hemagglutinin (FHA) and fimbriae are also highly expressed. In the avirulent Bvg^−^ phase, the virulence factors are not expressed (Mattoo & Cherry, 2005). Bvg modulations are thought to affect profiles of gene transcriptions in a gradual range of changes (Cummings *et al.*, 2006). Between Bvg^+^ and Bvg^−^ phases is at least one distinct phase named the Bvg intermediate phase (Bvg^i^) (Cotter & Miller, 1997), expressing a different set of gene products from Bvg^+^ and Bvg^−^ phases. Genes such as *bipA* are upregulated specific to the Bvg^i^ phase, as well as the adhesins such as FHA and fimbriae that are expressed at both Bvg^+^ and Bvg^i^ phases.

The effects of CyaA and type III secretion system on host cells are well-characterized and are the primary causes for *in vitro* cytotoxicity (Cotter *et al.*, 1998; Yuk *et al.*, 2000; Vojtova *et al.*, 2006). In addition, they interfere with the host immune systems to facilitate the successful infection of the host (Skinner *et al.*, 2004; Reissinger *et al.*, 2005; Vojtova *et al.*, 2006). FHA possesses at least four binding domains involved in attachment including the Arg–Gly–Asp (RGD) triplet, the leukocyte response integrin/integrin-associated protein (LRI/IAP) complex, the complement receptor type 3 (CR3) and the carbohydrate recognition domain (CRD) (Relman *et al.*, 1990; Saukkonen *et al.*, 1991; Prasad *et al.*, 1993; Ishibashi *et al.*, 1994). FHA has been characterized as the major adhesin that mediates binding to the cilia of the respiratory epithelial cells by the CRD domain (Prasad *et al.*, 1993). In addition, it was previously shown that FHA is largely responsible for mediating biofilm formation (Irie *et al.*, 2004).

A majority of the reported research on *Bordetella* pathogenesis has focused on the infection of the lower respiratory tract. However, the primary host reservoir for *B. bronchiseptica* colonization is thought to be the nasal cavity. Bvg phase regulation is sensitive to temperature (Cotter & Jones, 2003), with the Bvg^+^ phase being expressed at 37°C while the Bvg^−^ phase is predominant at 25°C and below. The mammalian mucosal temperature in the nasal cavity has been estimated to be ∼30°C (Jacky, 1980), which supports a Bvg^i^ phase growth (Williams *et al.*, 2005). In the Bvg^i^ phase, toxins are not expressed, but the adhesins are. We report in this study that FHA plays a major role in the colonization of the nasal cavity, and provide indirect evidence that the bacteria are in the Bvg^i^ phase in the nose.

## Materials and methods

### Bacterial strains and growth conditions

*Bordetella bronchiseptica* strains RB50 [wild type (WT)], RB53 (Bvg^+^ phase-locked mutant), RB53i (Bvg^i^ phase-locked mutant), and RBX9 (Δ*fhaB*) were previously reported and characterized (Cotter & Miller, 1994, 1997; Cotter *et al.*, 1998). *Bordetella bronchiseptica* strains were propagated in Stainer–Scholte (Stainer & Scholte, 1970) liquid medium or on BG agar (Becton Dickinson) supplemented with defibrinated sheep blood at 37°C.

### Animal colonization

High-dose intranasal infections of anesthetized mice and recovery of bacteria were performed as previously described (Harvill *et al.*, 1999) and at least three mice per experiment were used. C57/BL6 mice aged 6–10 weeks old were obtained from the National Cancer Institute and housed in insulator cages and cared for in accordance with Institutional Animal Care and Use Committee-approved protocols at the University of Pennsylvania School of Medicine animal facility. For tissue sectioning, sacrificed mice were bled by cardiac puncture before decapitation. For bacterial counts, CFU were determined by plating serially diluted homogenized lungs and vortexed trachea and nasal cavity tissue materials.

### Tissue sections

Soft tissues were surgically removed from the skulls of decapitated heads. The skulls were then immersed in 4% paraformaldehyde phosphate-buffered saline (PBS) solution for 24 h, gently washed with water, and decalcified in Decal solution (Decal Chemical Corp) for 24 h. Skulls were then imbedded in Tissue-Tek O.C.T. (Sakura) before freezing in 2-methylbutane immersed in liquid nitrogen. Samples were sectioned at ∼10 μm thickness with cryotome blades and mounted on glass microscopy slides.

### Immunofluorescence

Frozen sections were fixed in acetone at 4°C for 10 min. They were then rehydrated in PBS, incubated in 0.1% sodium borohydride for 15 min. Sera from three mice infected with WT *B. bronchiseptica* for 100 days were pooled after retro-orbital bleeding and centrifuging and used as primary antibodies for the stains. Secondary antibodies used were Alexa Fluor 594-conjugated goat anti-mouse IgG (Molecular Probes). Samples were incubated with each solution of antibodies diluted 1 : 500 in PBS for 30 min each at room temperature. After each incubation step, samples were washed three times with PBS.

### Hematoxylin & eosin (H&E) staining

Frozen sections were first washed in water for 3 min. They were then stained with hematoxylin solution (Surgipath) for 2 min. followed by a wash step under running tap water for 2 min. The slides were exposed to 50% ethanol for 1 min and then stained with eosin solution (Surgipath) for 1 min. The slides were rehydrated in alcohol ascending battery (from 70% to 100%) 1 min per step. Slides were then cleared with xyline (Surgipath) and a coverslip was applied using Permount medium (Fisher).

### Microscopy

All fluorescence images were taken using Improvision Open Lab software and a Leica DM R epifluorescence microscope. Deconvolution was performed on all images using Improvision Volocity.

## Results

The biology of *B. bronchiseptica* in the nasal cavities of infected hosts has not been extensively characterized. From previous reports, Bvg^−^ phase-locked mutants were completely deficient in colonization (Cotter & Miller, 1997), and Δ*fhaB* mutants (functional deletion of FHA) had lower CFU recovered from dissected nasal cavity tissue materials (Cotter *et al.*, 1998). To investigate colonization patterns throughout the nasal cavity, tissue sections were carried out at different intervals of the murine skulls along the anterial–posterial axis. The different section intervals, based on a previous histological description (Uraih & Maronpot, 1990), were examined according to structural hallmarks at each region of the nasal cavity. Level 1 is the most anterior region of the nasal cavity, closest to the nares. The most significant differences between the different levels are the types of epithelial cells lining the mucosa. The primary observation is that there is a significant transition from ciliated respiratory epithelia to the nonciliated olfactory epithelia qas progression is made into the nasal cavity toward the posterior end of the skull. The epithelial cell types of the specific nasal structures at various interval sections of the nasal cavity, as described by an earlier study (Uraih & Maronpot, 1990), are summarized in the first four columns of Table 1.

**Table 1:** Comparison of colonization profiles between WT and Δ*fhaB* mutant in the nasal cavity

Level	Location	Epithelia type	Ciliated?	WT colonization	Δ*fhaB* colonization
1	Septum	Respiratory	Yes	No	No
	Turbinates (naso- and maxillo-)	Respiratory	Yes	No	No
2	Septum	Respiratory	Yes	Yes	Yes
	Turbinates (naso-)	Transitional	Sparsely	Sparse	Sparse
2.5	Septum	Respiratory	Yes	Yes	Yes
	Turbinates (naso-)	Olfactory	No	Yes	No
3	Septum	Olfactory	No	Yes	No
	Turbinates (ethmoid)	Olfactory	No	Yes	No
4	Septum	Olfactory	No	Yes	No
	Turbinates (ethmoid)	Olfactory	No	Yes	No

### FHA mutants are unable to colonize nonciliated olfactory epithelia

**Fig. 1:**
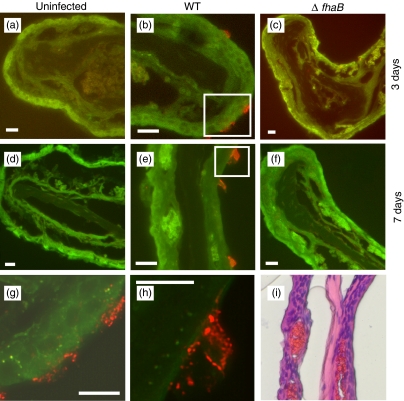
Immunofluorescence images of representative olfactory epithelia (Level 2.5 naso-turbinates). (a–f) Nasal cavity sections were prepared 3 and 7 days postinfection by WT or Δ*fhaB Bordetella bronchiseptica*. Only WT was able to colonize the olfactory epithelia. At 7 days postinfection, WT bacteria formed large three-dimensional structures (microcolonies). Tissues were stained with mouse anti-*B. bronchiseptica* antibodies. Red, *B. bronchiseptica*. Green, pseudo-coloured tissue autofluorescence. Magnification, × 200. Scale bars, 16.75 μm. (g–h) High-resolution images (magnification, × 630) of colonizing WT bacteria. Corresponding areas (g) and (h) are boxed in (b) and (e), respectively. Scale bars, 10 μm. (i) H&E stain of an adjacent section to (e).

**Fig. 2:**
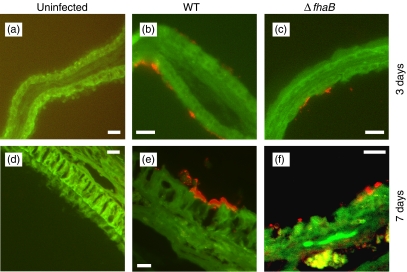
Immunofluorescence images of representative respiratory epithelia (Level 2.5 septum). Nasal cavity sections were prepared 3 (a–c) and 7 (d–f) days postinfection by WT or Δ*fhaB*. Both WT and Δ*fhaB* were able to colonize the respiratory epithelia. Tissues were stained with mouse anti-*Bordetella bronchiseptica* antibodies. Red=*B. bronchiseptica*. Green=pseudo-coloured tissue autofluorescence. Magnification, × 200. Scale bars, 16.75 μm.

Upon studying WT and Δ*fhaB*-infected murine nasal cavities by immunofluorescence microscopy of tissue sections, it was apparent that Δ*fhaB* mutants were incapable of colonizing areas of the nasal cavity that consist primarily of nonciliated olfactory epithelia (Table 1 and Fig. 1). Interestingly, all strains of *B. bronchiseptica* were absent at the most anterior end of the tissue sections (Level 1). The colonization profiles of WT and Δ*fhaB* bacteria on the respiratory and transitional epithelia at Level 2 and 2.5, however, were identical (Table 1 and Fig. 2). Colonizing WT bacteria ultimately grew into large three-dimensional structures after 7 days of infection (Fig. 1e). The enlarged image of one of these microcolonies clearly shows that this is a structured bacterial aggregate (Fig. 1h). By H&E stain of an adjacent tissue section (Fig. 1i), there appear to be no superficial damages to the host epithelia in the area of *B. bronchiseptica* colonization or any neutrophil infiltration that can contribute to the observed microcolony structures. Similar microcolony formations have been observed previously *in vitro* for *B. bronchiseptica* biofilms (Irie *et al.*, 2004). It is possible that the colonizing bacteria are growing in a biofilm-like state.

FHA of *Bordetella* has been characterized to have specific binding affinities to the cilia of mammalian respiratory cells (Smith *et al.*, 2001). The absence of Δ*fhaB* in the olfactory epithelia implicates that binding to these tissues *in vivo* is FHA-dependent but not cilia-mediated. FHA has also been shown to mediate binding to nonciliated cells such as macrophages (Saukkonen *et al.*, 1991), and possesses at least four independent binding domains involved in attachment (Mattoo & Cherry, 2005). As colonizations of the nasal respiratory epithelia by WT and Δ*fhaB* appear to be indistinguishable (Table 1 and Fig. 2), this suggests that the binding to these ciliated cell types is FHA-independent.

**Fig. 3:**
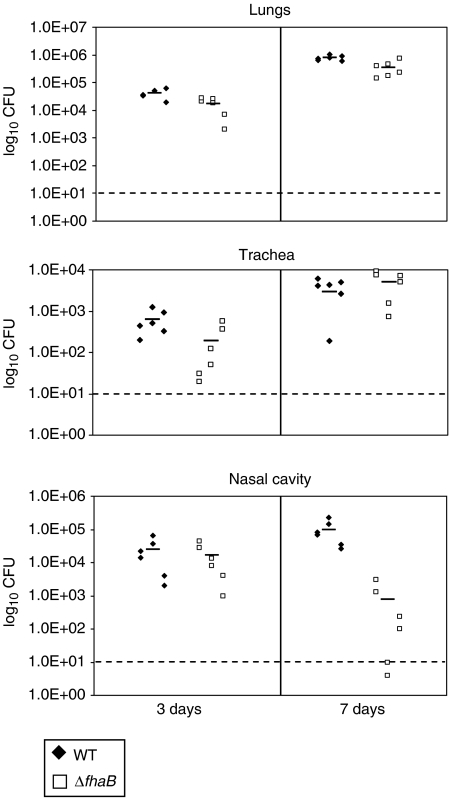
Δ*fhaB* has colonization deficiency in the nasal cavity at 7 days postinfection. Mice were infected with WT or Δ*fhaB* and sacrificed on the days indicated. Short horizontal bar represents mean recovered CFU. Dotted line represents the limit of detection. Presented figure is a representative of at least six independent experiments.

Further supporting the data observed by microscopy, the quantities of Δ*fhaB* mutants recovered from dissected nasal cavity tissue materials of mice were slightly less than those from WT-infected animals at 7 days postinfection (Fig. 3; *p*≈0.0126), consistent with previous studies carried out in rats (Cotter *et al.*, 1998). At this time point, significant numbers of microcolonies in WT-infected samples but none from samples infected by the Δ*fhaB* mutant could be observed. The Δ*fhaB* bacteria recovered from the nasal cavity probably represented those that were colonizing the nasal respiratory epithelium. It is unclear why at 3 days postinfection, WT and Δ*fhaB* mutants were recovered in similar CFU. It is possible that the enlargement of the microcolony structures is required to reflect differences in the log scale.

### Evidences for the expression of the Bvg^i^ phase *in vivo*

**Fig. 4:**
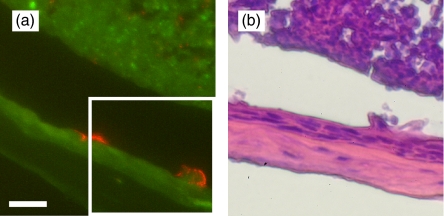
(a) Immunofluorescence image of naso-turbinate colonized with RB53, 7 days postinfection. Magnification, × 200. Scale bar, 16.75 μm. Corresponding H&E stain image (b) of an adjacent section is boxed. Unlike Fig. 1(i), the sites of bacterial colonization are marked with major histological alterations of the epithelium.

Bvg^i^ phase *in vitro* has previously been characterized as important for surface attachment and biofilm formation (Irie *et al.*, 2004). Biofilm formation is FHA dependent, leading one to investigate whether the *in vitro* phenotype is related to observed bacterial colonization *in vivo*, which is also FHA dependent and is in a microscopically biofilm-like state. The expression of the Bvg^i^ phase *in vivo* has been controversial for some time, and it has been unclear as to whether the Bvg^i^ phase is part of the pathogenic life cycle of *B. bronchiseptica* (Vergara-Irigaray *et al.*, 2005). The major challenge has been that BipA (function and *in vivo* expression unknown) is currently the only characterized gene product specific to the Bvg^i^ phase and not to the Bvg^+^ or Bvg^−^ phase (Stockbauer *et al.*, 2001). WT-infected nasal cavity tissue sections did not demonstrate the presence of BipA when they were stained with antibodies raised against BipA (data not shown). Since close examinations were not carried out for nasal cavity *B. bronchiseptica* colonization in the past, an attempt was made to investigate the specific Bvg phase the bacteria were expressing in the nasal cavity. From earlier studies, Bvg^−^ phase-locked strains are completely deficient in colonization of the host (Cotter & Miller, 1994) and there are strong evidences that Bvg^−^ phase is not expressed during colonization (Cotter & Miller, 1994; Akerley *et al.*, 1995). Mice were then infected with Bvg^+^ phaselocked mutants (RB53). From the immunofluorescence images (Fig. 4a), it seemed that this mutant could form microcolonies, but upon inspecting the H&E stain of an adjacent section, it was discovered that it was caused by major perturbations of the host epithelial tissue at the sites of colonized bacteria (Fig. 4b) instead of by threedimensional bacterial aggregates. Various toxins such as adenylate cyclase/hemolysin toxin and the type III secretion effector(s) such as BteA/BopC (Panina *et al.*, 2005; Kuwae *et al.*, 2006) that cause cytotoxicity to mammalian cells are only expressed in the Bvg^+^ phase. It is possible that the expression of such toxins by RB53 in the nasal cavity was causing such damage to the host tissue. Because such damage to the host tissue by WT bacteria was not observed (Fig. 1e and i), it is probable that WT *B. bronchiseptica* are not expressing the Bvg^+^ phase in the nasal cavity. In order to confirm whether colonized bacteria are expressing the Bvg^i^ phase, mice were also infected with Bvg^i^ phase-locked mutants (RB53i). However, consistent with previously published results, RB53i colonizes the respiratory tract including the nasal cavity poorly compared with WT (Cotter & Miller, 1997), indicating that intact Bvg phase switch mechanism is required for the successful maximum establishment of bacterial colonization. Because RB53i has been characterized to hyper-aggregate (Cotter & Miller, 1997; Stockbauer *et al.*, 2001), traditional CFU counts on solid media may be largely under-represented. However, there appeared to be fewer bacterial microcolonies in the nasal cavity compared with WT, complementing quantitative bacterial counts from dissected tissues (data not shown). Because colonization levels are different between RB53i and WT, direct comparison is difficult to accomplish, indicating that RB53i is not appropriate for *in vivo* studies of the Bvg^i^ phase. Despite this difficulty, RB53i found in the nasal cavity was found to form similar sizes of microcolony structures on the nasal mucosa (data not shown), indicating that the lower RB53i CFU was not due to a structural aggregation defect, but possibly due to insufficient surface inoculation coverage during infection, defect in initial attachment (highly unlikely, given the enhanced attachment properties of RB53i), defect in competition against other resident species on the nasal mucosa, and/or defect in survival of RB53i against host immune system.

Absence of *B. bronchiseptica* was observed at Level 1 sections, possibly due to the lower temperature of the surface mucosa near the nares, as the air is most likely representative of ambient temperature. At such temperature, *B. bronchiseptica* expresses the Bvg^−^ phase and may be incapable of attachment and other factors necessary to establish colonization, as *B. bronchiseptica* was also not seen infecting Level 1 sections at an earlier time point (Day 1 postinfection data not shown). Alternatively, *B. bronchiseptica* may be experiencing niche competition against commensal species heavily colonizing the areas of the nasal cavity closest to the outside.

## Discussion

Results from this study reveal interesting observations of *B. bronchiseptica* colonization of the nasal cavity, an under-researched site of *B. bronchiseptica* pathogenesis despite being widely believed to be the primary site of environmental reservoir. WT bacteria were observed to form three-dimensional microcolony aggregates on nasal epithelia, resembling *in vitro* biofilm microcolonies (Irie *et al.*, 2004). While WT bacteria were able to colonize ciliated respiratory epithelia and nonciliated olfactory epithelia, FHA mutants were only able to colonize ciliated respiratory epithelia. This result indicates that FHA is an important factor for adhesion to nonciliated epithelia, but adhesion to ciliated epithelia is FHA independent. FHA has been associated with cilia adhesion in previous publications (Smith *et al.*, 2001), but all studies have been carried out with tracheal ciliated epithelia cells (Edwards *et al.*, 2005). Based on the discrepancies between the results described in this report and previous publications, it is highly likely that there are physiological differences between the cilia found in the nasal cavity and the trachea. However, no publications could be found comparing the two, as there are very few studies focusing on cell biology of the nasal cavity epithelia. FHA adhesion to ciliated nasal epithelial cells (not exclusively to cilia themselves) has been suggested previously (Register & Ackermann, 1997), but *in vitro* data on cultured nasal epithelia might not be completely representative of *in vivo* host–pathogen interactions.

FHA-dependent aggregation and adhesion have been demonstrated for biofilm formation *in vitro*. Because maximal biofilm formation has been observed when *B. bronchiseptica* is expressing the Bvg^i^ phase (Irie *et al.*, 2004), and the bacterial microcolonies found on the nasal epithelial cells closely resembled biofilm microcolonies, it was next examined whether *B. bronchiseptica* are expressing the Bvg^i^ phase in the nasal cavity *in vivo*. Bvg^i^ phase-locked mutants have colonization deficiency, indicating the importance of intact Bvg phase variation mechanisms for proper bacterial colonization in the respiratory tract, most likely very early postinoculation in the infective cycle of the pathogen. Furthermore, antibodies raised against the only characterized Bvg^i^ phase-specific marker protein, BipA, failed to react with bacterial aggregates observed in the nasal cavity tissue sections. BipA expression *in vivo* has been investigated in previous studies, but definitive evidence for its expression has not been demonstrated (Vergara-Irigaray *et al.*, 2005). It is therefore still unclear whether BipA can be used as an appropriate Bvg^i^ phase marker for *B. bronchiseptica* found *in vivo*. Lacking tools for direct Bvg^i^ phase examination, inspections focusing on Bvg^−^ and Bvg^+^ phases were imperative. Consistent with previous publications from other groups, Bvg^−^ phase-locked mutants are completely deficient in establishing colonization in the respiratory tract. Bvg^+^ phase-locked mutants appeared to colonize the nasal cavity similar to WT, but close examination of infected tissues revealed major alterations of the epithelial lining, as WT-infected tissues were indistinguishable with uninfected epithelia. From these results, it can be concluded that *B. bronchiseptica* found in the nasal cavity are expressing neither Bvg^+^ nor Bvg^−^ phases, and therefore indirectly suggests the expression of the Bvg^i^ phase. However, the possibility that *B. bronchiseptica* are expressing an entirely different Bvg phase *in vivo* (Bvg^*vivo*^ phase) from the Bvg phase variation continuum well-described from *in vitro* studies cannot be excluded. Bvg^*vivo*^ is likely to be BvgAS dependent, and have a ‘rheostat-like gradual control,’ similar to Bvg phase variation observed *in vitro*.

If *B. bronchiseptica* are growing in a biofilm-like state in the nasal cavity, bacteria may be detaching from nasal epithelia as part of a biofilm developmental life cycle. Many *in vitro* biofilm systems have been observed to cycle through steps of surface attachment, biofilm formation and development, and detachment of a smaller population of cells from the main biofilm body (Hall-Stoodley & Stoodley, 2005). *Bordetella bronchiseptica* biofilm development can also be envisioned to follow similar life cycles. The only proposed roles of Bvg^i^ phase has been attributed to transmission of *Bordetella* from one host to another (Cotter & Miller, 1997), and such detachment may contribute to transmission if bacteria exit the respiratory tract, or to the persistent chronic nature of *B. bronchiseptica* infection if bacteria are inhaled into the lower respiratory tract (Irie *et al.*, 2004). Adhesion to cilia in the nasal cavity may also be critical for bacterial spread within and across hosts since the maximum fluid flow dynamics can be expected on ciliated surfaces, and shear force has been shown to play a significant role in biofilm detachment and dispersion (Hall-Stoodley & Stoodley, 2005).

Nasal mucosal surface has unique properties that make drug treatments less effective, such as lower diffusion rates (Donovan *et al.*, 1990) and rapid mucociliary clearance (Schipper *et al.*, 1991). Clearance of chronic persistent pathogenic species colonizing the nasal cavity is a medically challenging future issue. Further studies of *B. bronchiseptica* in the nose will benefit in better understanding of long-term colonizations of pathogens in the hosts.

## Statement

Re-use of this article is permitted in accordance with the Creative Commons Deed, Attribution 2.5, which does not permit commercial exploitation.
